# Objective Pelvic Support and Patient-Reported Outcomes After Non-Ablative Vaginal Er:YAG Laser Therapy in Women with Pelvic Floor Dysfunction: A Prospective Single-Arm Interventional Study in a Real-World Care Setting

**DOI:** 10.3390/healthcare14081021

**Published:** 2026-04-13

**Authors:** Laia Blanco-Ratto, Montserrat Girabent Farrés, Cristina Naranjo Ortiz, Stephanie Kauffmann, Manuel Del Campo Rodríguez, Inés Ramírez-García

**Affiliations:** 1 RAPbarcelona Physiotherapy Clinical Center, 08037 Barcelona, Spain; laia.blanco@rapbarcelona.com (L.B.-R.); stephanie.kauffmann@rapbarcelona.com (S.K.); 2 Doctoral Programme in Health, Wellbeing and Bioethics, Universitat Ramon Llull, 08025 Barcelona, Spain; 3 Campus Docent Sant Joan de Déu, Universitat de Vic-Universitat Central de Catalunya (UVic-UCC), 08500 Barcelona, Spain; montserrat.girabent@sjd.edu.es; 4 Yale Cancer Center, Yale School of Medicine, Yale University, New Haven, CT 06520, USA; cnaranjoortiz@gmail.com; 5 Teknon Medical Center, 08022 Barcelona, Spain; ginedelcampo@gmail.com; 6 Blanquerna School of Health Sciences, Universitat Ramon Llull, 08025 Barcelona, Spain; 7 GHenderS Research Group, Blanquerna School of Health Sciences, Universitat Ramon Llull, 08022 Barcelona, Spain

**Keywords:** non-ablative Er:YAG laser, non-ablative laser, minimally invasive therapy, pelvic floor dysfunction, pelvic organ prolapse, female sexual function, patient-centered care, energy-based devices

## Abstract

**Highlights:**

**What are the main findings?**

- This experimental non-ablative Er:YAG laser intervention produced measurable short-term anatomical improvements in pelvic floor support using standardized POP-Q assessment in a real-world clinical setting.
- Anatomical changes occurred without parallel improvement in global sexual function, highlighting a potential dissociation between structural remodeling and early functional outcomes.

**What are the implications of the main findings?**

- These results contribute objective data to the ongoing debate on energy-based interventions, supporting their evaluation as minimally invasive outpatient options for selected women with pelvic floor dysfunction.
- The study underscores the need for controlled trials to determine whether anatomical remodeling translates into sustained functional benefit and improved patient-centered outcomes.

**Abstract:**

**Background/Objectives**: Non-ablative vaginal Erbium-doped Yttrium Aluminium Garnet (Er:YAG) laser therapy has been proposed as a minimally invasive option for pelvic floor dysfunction (PFD), yet objective anatomical data using standardized measures remain limited. This study evaluated short-term anatomical and functional outcomes in a real-world care setting. **Methods**: This prospective single-arm interventional cohort study included women with PFD who underwent two sessions of non-ablative vaginal Er:YAG laser therapy. Outcomes were assessed at baseline, first follow-up (FU1), and second follow-up (FU2). Anatomical changes were measured using POP-Q parameters, including vaginal hiatus (Gh), total vaginal length (TVL), and compartmental staging. Sexual function was evaluated using the Female Sexual Function Index (FSFI). Pelvic floor muscle strength was assessed using the Oxford Scale. Non-parametric tests were used for repeated measures, and correlations between delivered laser energy and clinical outcomes were explored. **Results**: A total of 163 women were enrolled; 136 completed FU1 and 59 completed FU2. Median vaginal hiatus decreased significantly from baseline to FU1 and remained reduced at FU2 (*p* < 0.001). Improvements in anterior and posterior prolapse staging were observed, with a shift toward lower POP-Q stages at both follow-up visits. FSFI total scores did not change significantly across visits, although small changes were observed in specific domains, including a transient decrease in orgasms at FU1 (Δ = −0.2, *p* = 0.021) and a modest improvement in pain at FU2 (Δ = −0.4, *p* = 0.045). The magnitude of anatomical changes was modest, and their clinical relevance remains uncertain. **Conclusions**: Non-ablative vaginal Er:YAG laser therapy was associated with short-term improvements in vaginal hiatus and POP-Q prolapse staging in women with PFD, while sexual function remained stable. These findings provide objective anatomical data on early treatment effects in routine care, informing future evaluation of minimally invasive care models for pelvic floor dysfunction.

## 1. Introduction

Pelvic floor dysfunction (PFD) encompasses pelvic organ prolapse (POP), urinary incontinence, pelvic pain, and sexual dysfunction, and substantially impairs women’s physical, psychological, and sexual well-being. PFD affects approximately one-third of women and is associated with diminished quality of life, including increased psychological distress and reduced sexual well-being [1,2,3,4].

Several risk factors are known to contribute to the development of PFD, with advanced age, high body mass index (BMI), menopause, and multiparity being the most relevant [5,6]. Conservative options such as pelvic floor muscle training, pessaries, and behavioral therapies, as well as surgical approaches, are available; however, each has limitations related to adherence, durability, or invasiveness [7,8].

In recent years, non-ablative vaginal laser therapy has emerged as a minimally invasive alternative for treating various pelvic floor disorders. Energy-based devices, including fractional CO_2_ and Erbium-doped Yttrium Aluminium Garnet (Er:YAG) lasers, aim to induce controlled thermal stimulation to promote re-epithelialization, neovascularization, and collagen remodeling. Reported benefits include improvements in genitourinary syndrome of menopause, vaginal laxity, and stress urinary incontinence [9,10,11,12].

Despite growing interest, international societies such as ACOG and EUGA emphasize the investigational nature of laser therapy, citing a lack of randomized trials, small sample sizes, limited use of objective anatomical endpoints, and short follow-up periods [9,10,11,12,13].

In this context, we conducted a prospective single-arm interventional cohort study in a real-world clinical setting, in which women with PFD who were undergoing routine care received two sessions of non-ablative vaginal Er:YAG laser therapy, and clinical data were collected and analyzed longitudinally to characterize short-term anatomical and functional changes following non-ablative vaginal Er:YAG laser therapy. Specifically, we assessed changes in vaginal hiatus and total vaginal length using standardized physical examination techniques, along with changes in sexual function as measured by the validated Female Sexual Function Index (FSFI) questionnaire [14]. Although a single-arm design does not allow causal inference, it enables structured characterization of short-term treatment-associated changes in a real-world clinical population and provides objective POP-Q-based measurements that remain underreported in previous studies. We hypothesized that non-ablative vaginal Er:YAG laser therapy would be associated with short-term improvements in objective anatomical parameters without corresponding changes in global sexual function. Additionally, regulatory agencies have raised concerns regarding the use of energy-based devices for gynecologic indications, highlighting the need for cautious interpretation of emerging evidence.

## 2. Materials and Methods

A prospective single-arm interventional cohort was undertaken to assess anatomical and functional changes in women with PFD treated with non-ablative vaginal laser therapy (Fotona, d.o.o., Ljubljana, Slovenia). Recruitment occurred from January 2024 to October 2024 at a specialized urogynecology unit in Barcelona, Spain. The study was registered under ISRCTN 11575756. Ethics committee approval was obtained from the Blanquerna CER Committee under approval number 2024-01-01 on 24 January 2024, and conducted in accordance with the Declaration of Helsinki and applicable data protection regulations. All participants provided written informed consent.

A convenience sample was used: 268 women were assessed for eligibility, and 163 met all inclusion criteria and were enrolled. Women were eligible if they self-reported PFD symptoms (urinary incontinence, pelvic organ prolapse, or sexual dysfunction) and perceived vaginal laxity, defined as Vaginal Laxity Questionnaire (VLQ) ≥ 4; additional requirements were the ability to complete validated questionnaires and authorization for use of anonymized clinical data. Exclusion criteria were previous vaginal laser therapy, contraindications to non-ablative vaginal laser, and concomitant interventions expected to confound outcomes during the evaluation period (e.g., pelvic floor physiotherapy, vaginal radiofrequency, or other concurrent treatments). Participants were advised not to initiate or modify concomitant PFD treatments during follow-up, although such changes were not systematically recorded. This represents a potential source of confounding, particularly in a real-world setting. No formal sample size calculation was performed, as this study was designed as an exploratory real-world cohort. Missing data were not imputed; analyses were conducted using complete cases for global comparisons and available pairs for pairwise analyses. Data were analyzed using SPSS Statistics (v26.0).

The intervention consisted of IncontiLase^®^ and IntimaLase^®^ protocols delivered with the SP DYNAMIS system (S/N 14003339), aiming for controlled mucosal heating to approximately 65 °C to promote tissue tightening and collagen remodeling. Two sessions were administered 6–8 weeks apart by experienced operators; analgesia was provided as needed, eye protection and antiseptic preparation were used, and standard post-procedure instructions were given. The total pulse energy delivered to the anterior wall, posterior wall, 360° region, and vulvar region was recorded per session (Table 1). Safety was assessed clinically at each treatment and follow-up visit; transient post-procedural sensations (local warmth, mild stinging, watery discharge, transient vulvovaginal erythema/edema) were recorded separately from adverse events (persistent pain, dysuria, vaginal spotting, irritation, or vaginal/urinary tract infection). This structured approach ensured consistent documentation of both energy parameters and short-term safety signals.

Primary anatomical outcomes were vaginal hiatus and total vaginal length. Vaginal hiatus was assessed following the criteria set forth by the joint report of the International Continence Society and the International Urogynecological Association (ICS/IUGA) on terminology for female pelvic floor dysfunction [15]. Total vaginal length was defined as the distance in centimeters from the posterior fornix to the hymen, with point C or D reduced to its normal position [16]. Pelvic floor muscle strength was assessed by vaginal palpation using the Oxford scale, and sexual function was evaluated with the self-administered Female Sexual Function Index (FSFI), which assesses six domains (desire, arousal, lubrication, orgasm, satisfaction, pain) [14].

The FSFI total score was selected a priori as the primary functional endpoint, as recommended in prior psychometric evaluations.

All POP-Q points (Aa, Ba, C, D, Ap, Bp, Gh, Pb, TVL) were recorded at each visit in the lithotomy position during maximal Valsalva using a millimetric ruler, in accordance with ICS/IUGA terminology and measurement standards [15]. Before examination, the bladder and rectum were emptied, and a Sims speculum was used to retract the vaginal walls to optimize visualization [17]. Prolapse was clinically categorized as cystocele, uterine/apical prolapse, or rectocele, and severity was staged with POP-Q; for descriptive analyses, stages I–IV were summarized by the compartment with the greatest descent. When feasible, the same trained assessor examined each participant across visits. Because neither assessors nor participants could be blinded to visit timing, strict adherence to standardized examination procedures was emphasized to reduce measurement variability and minimize potential detection bias.

Assessments were performed at baseline before the first laser session (BE, *n* = 163), at first follow-up 6–8 weeks after the first session (FU1, *n* = 136), and at second follow-up 6–8 weeks after the second session (FU2, *n* = 59). Losses to follow-up were attributed to scheduling constraints, relocation, intercurrent illness (including pregnancy), voluntary withdrawal of consent, or changes in concomitant pelvic floor treatments; reasons were documented when available.

For statistical analysis, the Friedman test was used to compare BE, FU1, and FU2, reporting the global effect size as Kendall’s W. Pairwise contrasts (BE–FU1, BE–FU2, FU1–FU2) used the Wilcoxon signed-rank test, reporting the Hodges–Lehmann estimator (Δ) with 95% confidence intervals and the effect size r = |Z|/√N_pairs; *p*-values for pairwise comparisons were adjusted using Holm’s procedure. Analyses of the six FSFI domains were designated as secondary outcomes, with Holm-adjustment applied within each family of tests. Friedman tests were performed on complete cases, whereas pairwise tests were conducted on all available pairs. A significance level of α = 0.05 was established. Analyses were performed in IBM SPSS Statistics, version 26, and Stata, version 17.

## 3. Results

A total of 163 women were enrolled in the study. The mean age at baseline was 46.9 years and the mean BMI was 22.8 kg/m^2^. Most participants (82.5%) reported one or two full-term births, while 19.5% had a history of miscarriage. Urinary incontinence was documented in 55.8% of the cohort, and 81.6% presented some degree of pelvic organ prolapse, most frequently cystocele (80.4%). More than half of participants (58.1%) received both IntimaLase^®^ and IncontiLase^®^ protocols (Table 2). The total energy delivered was higher during the first treatment session across intravaginal and vulvar regions (Table 1), reflecting standard clinical practice in this protocol. Non-ablative vaginal Er:YAG laser therapy was well tolerated. The most common transient sensations were local warmth, mild stinging, and a short-lasting increase in vaginal discharge. These findings were self-limiting, required no medical intervention, and were not classified as adverse events. No clinically relevant adverse events were observed. In particular, there were no cases of significant bleeding, persistent pain, infection, or thermal injury. Overall, the treatment demonstrated an excellent safety profile in this real-world population.

### 3.1. Vaginal Hiatus and Vaginal Length

The vaginal hiatus significantly decreased over time (Kendall’s W = 0.533, *p* < 0.001) (Table 3). The median value decreased from 3 cm [2,3,4] at baseline to 2 cm [1,2,3] at first follow-up (Δ = –0.75, 95% CI: –0.5 to –0.75; *p* = 0.003), and this effect was maintained at the second follow-up with no further change (Δ = –0.5; *p* = 0.727). Although modest in magnitude, this reduction may reflect a short-term anatomical change in uncertain clinical significance, consistent with the proposed collagen remodeling mechanism. However, the proportion of participants achieving clinically meaningful improvement could not be determined, as predefined thresholds for minimal clinically important differences were not available.

**Figure 1:**
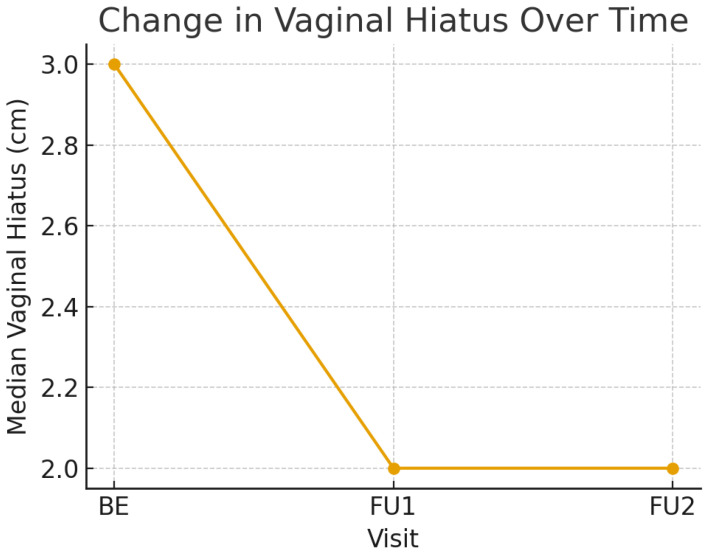
Change in vaginal hiatus over time. Median vaginal hiatus (cm) measured at baseline (BE), first follow-up (FU1), and second follow-up (FU2). Values displayed represent median measurements from POP-Q examination.

The clinical significance of this anatomical change (Figure 1) cannot be fully determined in the absence of prolapse-specific symptom measures. Total vaginal length remained stable across all visits. Although the Friedman test reached significance (*p* = 0.007; W = 0.031), pairwise comparisons revealed no consistent differences (all *p* > 0.05), with the median values maintained at 9 cm [9,10]. This stability aligns with the expected mechanism of the non-ablative Er:YAG laser, which is not designed to alter canal length.

### 3.2. Sexual Function

The total FSFI score did not show statistically significant variation throughout the follow-ups (Kendall’s W = 0.008, *p* = 0.628) (Table 4). Global sexual function therefore remained stable throughout follow-up. Among FSFI domains, arousal and satisfaction showed no significant changes (*p* = 0.062 and *p* = 0.259, respectively). A slight but statistically significant reduction in orgasm was identified at FU1 compared to baseline (Δ = −0.2, 95% CI: 0–0.2, *p* = 0.021), although this effect was not present at FU2. Regarding pain, a modest reduction was detected from baseline to FU2 (Δ = 0.4, 95% CI: 0–0.4, *p* = 0.045), suggesting potential improvement in dyspareunia for some participants. However, these domain-level changes were small in effect size and exploratory in nature. No significant changes were observed in desire, lubrication, or other domains. Overall, FSFI findings indicate stability in global sexual function despite anatomical changes.

### 3.3. Pelvic Floor Strength and Prolapse Staging

**Figure 2:**
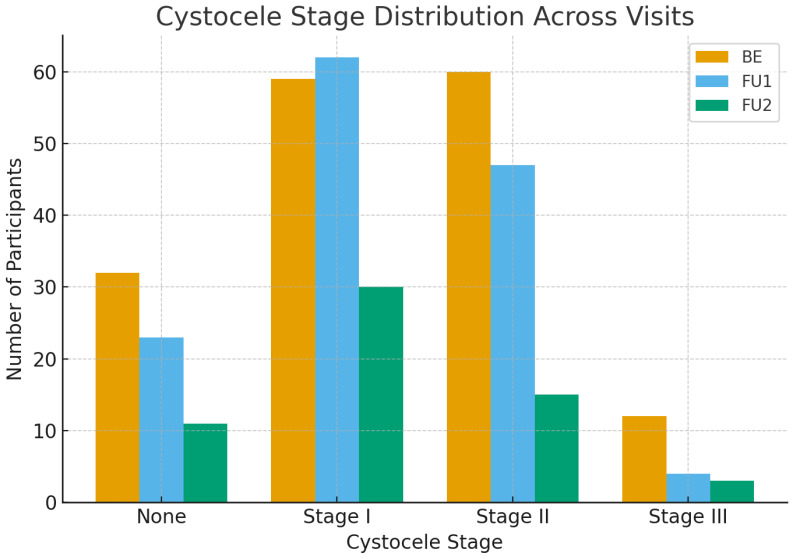
Cystocele stage distribution across visits. Distribution of cystocele severity at baseline (BE), first follow-up (FU1), and second follow-up (FU2). POP-Q stages are shown as proportions of the cohort at each visit.

As shown in Table 5, no statistically significant variation was observed in pelvic floor strength measured by the Oxford Scale (Kendall’s W = 0.048, *p* = 0.135). This stability may reflect the limited sensitivity of digital palpation and the fact that laser therapy primarily targets mucosal and connective-tissue remodeling rather than muscle hypertrophy. In contrast, significant improvements were observed in the anterior compartment prolapse (cystocele) (Kendall’s W = 0.164, *p* < 0.001). The proportion of women moving from Stage II to Stage I or no prolapse increased over time, suggesting improved support in the anterior compartment. Pairwise comparisons confirmed significant differences between BE and both follow-up visits (BE vs. FU1: *p* < 0.001, r = 0.375; BE vs. FU2: *p* = 0.001, r = 0.447), as well as between FU1 and FU2 (*p* = 0.013, r = 0.323), indicating that improvements were both early and sustained (Figure 2).

Although slight variation occurred in uterine prolapse stages (Kendall’s W = 0.037, *p* = 0.115), only the BE-FU1 comparison reached significance (*p* = 0.043, r = 0.210), with a small effect size and no consistent differences across follow-up, suggesting limited clinical significance. Rectocele staging demonstrated significant improvements (Kendall’s W = 0.166, *p* < 0.001), with more participants reporting no rectocele and fewer classified in Stage II or III. Significant BE-FU1 and BE-FU2 differences (BE vs. FU1: *p* < 0.001, r = 0.329; BE vs. FU2: *p* = 0.001, r = 0.456) suggest that posterior compartment support may also improve following treatment. The FU1-FU2 comparison approached significance (*p* = 0.058, r = 0.247), indicating a possible trend toward further anatomical benefit.

### 3.4. Association Between Energy Delivery and Anatomical/Functional Outcomes

A moderate correlation was identified between the energy applied to the anterior vaginal wall during the first session and reduction in vaginal hiatus diameter (r = 0.410, *p* < 0.001) (Table 6). Weak but statistically significant associations were also observed between energy applied to the 360° and vulvar regions during the second session and slight increases in vaginal length (r = 0.224 and 0.264; *p* = 0.008 and 0.003, respectively). All correlations with FSFI total score were non-significant, suggesting that sexual function stability was not directly related to energy parameters. Taken together, these findings indicate that anatomical changes—particularly in the anterior compartment—may be influenced by delivered energy, whereas functional sexual outcomes appear independent of dose parameters.

## 4. Discussion

This study assessed the clinical effects of non-ablative vaginal laser therapy in women with PFD, revealing statistically significant changes in prolapse stage and vaginal hiatus width, yet no statistically significant change in overall sexual function based on total FSFI scores. Although anatomical changes were observed, interpretation of later follow-up findings should remain cautious, given the reduced number of participants completing the final assessment.

The observed reduction in vaginal hiatus after laser therapy is consistent with the hypothesis that collagen remodeling induced by non-ablative Er:YAG laser may enhance vaginal support, although this mechanism remains speculative in the absence of direct histological confirmation. This is consistent with Long et al., who reported reduced vaginal cross-sectional area and improved tone on 3D transperineal ultrasound following similar treatment [18]. Blaganje et al. also reported improvements in pelvic floor contractility with objective perineometry, suggesting a potential structural effect on vaginal connective tissue [19]. Despite anatomical changes, vaginal length remained stable, a finding that parallels Lin et al., who observed no change in canal length but did describe reduced bladder neck mobility, suggesting enhanced pelvic support rather than modification of anatomical length [20].

Regarding sexual function, the total FSFI score did not change significantly after treatment. These results parallel previous studies, such as Long et al., who reported limited changes in global FSFI despite anatomical improvement [18]. Tien et al. noted perceived improvement among women and partners despite minimal urodynamic change, underscoring the complexity of relating anatomical and functional outcomes [21]. Minor improvements in pain resemble findings from Gaspar et al., who described reductions in genitourinary discomfort following laser therapy [22].

Prolapse staging improved after treatment, particularly in the anterior and posterior compartments. These findings are consistent with Kuszka et al., who described reduced prolapse severity and symptom improvement after repeated IncontiLase^®^ treatments [23]. The biological plausibility of this effect is supported by Shao et al., who associated BMI extremes with levator ani dysfunction, suggesting that tissue quality may influence responsiveness to connective-tissue-targeted therapies [24].

While comparison with surgical approaches for pelvic organ prolapse may appear relevant, direct comparison in this context is methodologically challenging and may be misleading. Surgical interventions and non-ablative laser therapy differ substantially in mechanism of action, invasiveness, patient selection, and expected magnitude of effect. The findings of this study should therefore be interpreted within the context of minimally invasive, non-surgical management rather than as comparable to established surgical procedures.

Laser-induced collagen remodeling may help explain the observed anatomical improvements, although the impact on pelvic floor muscle function remains less clear. Pelvic floor strength did not change significantly according to the Oxford scale, a subjective assessment method. This differs from the findings of Blaganje et al., who used perineometry, suggesting that more sensitive quantitative instruments may be required to detect small changes [19].

The distribution of risk factors in this cohort, including parity, BMI, and age, aligns with previous epidemiological studies identifying these variables as contributors to PFD [1,25,26,27].

Anatomically, prior work by Berger et al. demonstrated that levator ani defects are more closely associated with anterior compartment prolapse [27]. Our findings of anterior compartment improvement without measurable muscle strength change may therefore reflect selective effects on connective tissue rather than pelvic musculature. This is further supported by Karjalainen et al., who found that defecatory symptoms improved after posterior prolapse correction, suggesting that structural interventions can relieve symptoms without altering baseline muscular function [28].

Additionally, pulse energy correlated positively with hiatus reduction, suggesting a possible dose–response relationship consistent with the mechanism of collagen remodeling. However, no association was observed with total FSFI scores, indicating that short-term sexual function outcomes may not be energy-dependent. This aligns with Kuszka et al., who suggested that outcomes may depend on treatment frequency, with multiple sessions potentially yielding greater effect [23]. Sikora et al. also support vaginal laser as a minimally invasive alternative for young women between childbirths and postmenopausal patients, emphasizing its safety profile, outpatient feasibility, and the absence of synthetic materials [29].

This study has limitations that should be considered. The single-arm design precludes causal inference and limits interpretation to associations rather than treatment effects. With only 37% of participants completing the final follow-up, the possibility of attrition bias represents a major limitation, as participants who remained may differ systematically from those lost to follow-up. Although baseline characteristics were comparable, unmeasured differences could have influenced outcome estimates, particularly at later timepoints; the results should therefore be interpreted as exploratory. Additionally, the lack of systematic recording of concomitant treatments introduces potential confounding, particularly in a real-world cohort where physiotherapy or behavioral changes may influence anatomical outcomes. Clinical examination tools may also be less sensitive than instrumental measures, and condition-specific patient-reported outcomes (e.g., prolapse-related bother or incontinence severity) were not collected. Follow-up was limited to early post-treatment intervals and may not capture longer-term durability.

Despite these limitations, the study adds to the existing literature by providing standardized, objective POP-Q-based anatomical data following non-ablative Er:YAG laser therapy in a real-world clinical setting. While prior studies on vaginal laser interventions have primarily focused on symptom-based outcomes or specific conditions such as stress urinary incontinence or genitourinary syndrome of menopause, objective quantification of pelvic organ support using validated anatomical measures remains limited. In addition, this study integrates anatomical, functional, and energy-delivery data within a prospective cohort design, offering a more comprehensive characterization of early treatment-associated changes in routine clinical practice. These features differentiate our findings from previous reports and contribute novel data to an area where objective and clinically contextualized evidence is still emerging.

However, these findings should be interpreted within the broader context of ongoing debate regarding vaginal laser therapies. Regulatory agencies, including the U.S. Food and Drug Administration (FDA), have issued warnings highlighting the lack of robust evidence supporting the safety and effectiveness of energy-based devices for gynecological indications [13]. Likewise, professional societies such as ACOG and EUGA emphasize [9,13] that current evidence remains limited by small sample sizes, heterogeneity in outcome measures, and a predominance of uncontrolled study designs, with systematic reviews reporting inconsistent results and, in some cases, limited or no significant benefit compared to sham or standard care. In this context, our findings contribute to an emerging but still inconclusive body of evidence and should be considered preliminary, supporting the need for well-designed randomized trials with longer follow-up and comprehensive patient-reported outcomes to establish clinical effectiveness and durability. From an implementation perspective, these findings may inform the design of pragmatic trials and care pathways for minimally invasive, outpatient interventions for pelvic floor dysfunction, particularly within primary care and women’s health services.

## 5. Conclusions

Non-ablative vaginal Er:YAG laser therapy was associated with modest short-term improvements in vaginal hiatus measurements and prolapse stage, while sexual function and pelvic floor strength remained stable. These findings are relevant for primary care and women’s health services, where pelvic floor symptoms are commonly first identified and managed longitudinally. Minimally invasive interventions delivered in outpatient settings may represent an emerging care model for selected patients who are not candidates for or who decline surgical management, highlighting the need for implementation research and care pathway integration. Further research should include randomized controlled trials with longer follow-up to establish clinically meaningful outcome measures.

## Figures and Tables

**Table 1:** Pulse energy delivered during the first and second laser sessions.

Pulse Energy (Joules)	First Session		Second Session	
	Mean	SD	Mean	SD
Anterior	3827.66	2417	3454.87	1469.59
Posterior	1747.04	326.66	1602.88	511.17
360°	1092.83	348.09	1085.6	580.42
Vulvar region	15,902.12	7563.96	14,748.55	6436.17

Data are presented as mean (SD). Laser energy was recorded for each anatomical region during both sessions. J = joules.

**Table 2:** Baseline and demographic characteristics of participants (*n* = 163).

	*n*	%
Age *	46.86	8.89
Full-term births		
1–2	127	82.47
3–4	20	12.99
Preterm births	1	0.65
Miscarriages	30	19.48
Body Mass Index *	22.83	4.35
Urinary incontinence ^1^		
Yes	91	55.83
No	72	44.17
Prolapse		
Yes	133	81.60
No	30	18.40
Type of prolapse		
Cystocele	131	80.37
Uterine prolapse	40	24.54
Rectocele	111	68.10
Laser applied		
IncontiLase	32	20.00
IntimaLase	35	21.88
Both	93	58.13
Total	163	100.00

Values are presented as *n* (%) unless otherwise indicated. * Mean ± SD. ^1^ Reported as a symptom.

**Table 3:** Changes in vaginal hiatus and total vaginal length across follow-up visits.

	BE	FU1	FU2			BE vs. FU1			BE vs. FU2			FU1 vs. FU2		
	Med [p25–p75]	Med [p25–p75]	Med [p25–p75]	Global †	W	Δ (95% CI)	r	*p * *	Δ (95% CI)	r	*p * *	Δ (95% CI)	r	*p * *
Vaginal hiatus (cm)	3 [2–4]	2 [1–3]	2 [1–3]	<0.001	0.533	−0.75 (−0.5; −0.75)	0.773	0.003	−0.5 (−0.5; −0.75)	0.727	0.003	None	0.002	0.783
Vaginal length (cm)	9 [9,10]	9 [9,10]	9 [9,10]	0.007	0.031	None	0.221	0.063	None	0.156	0.282	None	0.290	0.726

Values are presented as median [p25–p75]. BE: baseline evaluation; FU1: first follow-up; FU2: second follow-up. † Friedman test; * Wilcoxon signed-rank test with Holm adjustment; Δ: Hodges–Lehmann estimator; r: effect size; W: Kendall’s W.

**Table 4:** Female Sexual Function Index (FSFI) scores at baseline and follow-up.

Female Sexual Function Index	BE		FU1		FU2		Global †	W	BE vs. FU1			BE vs. FU2			FU1 vs. FU2		
	Med [p25–p75]		Med [p25–p75]		Med [p25–p75]				Δ (95% CI)	r	*p * *	Δ (95% CI)	r	*p * *	Δ (95% CI)	r	*p * *
Total score	17.3	[14.5–19.8]	17.6	[15.65–19.15]	17.2	[15.8–19]	0.628	0.008	0.3 (1.05; −0.4)	0.097	0.777	None	0.004	0.985	−0.3 (NR)	0.120	0.777
Desire	2.4	[1.8–3.6]	2.4	[1.2–3.6]	2.4	[1.2–2.4]	0.005	0.092	−0.3 (0; −0.3)	0.102	0.059	−0.3 (NR)	0.307	0.009	−0.3 (NR)	0.334	0.024
Arousal	2.4	[1.8–3.6]	2.1	[1.8–3.3]	2.1	[1.5–2.7]	0.062	0.049	−0.15 (0; −0.3)	0.063	0.236	−0.15 (NR)	0.191	0.094	−0.3 (NR)	0.308	0.060
Lubrication	3.6	[3–3.6]	3.6	[3.3–3.9]	3.6	[3.3–3.6]	0.510	0.012	0.15 (0.3; 0)	0.233	0.081	0.15 (NR)	0.215	0.400	None	0.120	0.400
Orgasm	3.2	[2.8–3.6]	2.8	[2.8–3.6]	3.2	[2.8–3.6]	0.794	0.004	−0.2 (0; −0.2)	0.294	0.021	None	0.222	0.548	None	0.016	0.907
Satisfaction	1.6	[0.8–2.8]	1.2	[0.8–2.4]	1.6	[1.2–2]	0.259	0.024	None	0.190	0.423	None	0.125	1.000	None	0.109	1.000
Pain	5.2	[3.2–6]	5.2	[3.6–6]	5.6	[4.8–6]	0.117	0.038	0 (0.2; 0)	0.022	0.755	0.4 (NR)	0.242	0.045	0.2 (NR)	0.251	0.082

Values are presented as median [p25–p75]. BE: baseline evaluation; FU1: first follow-up; FU2: second follow-up. † Friedman test (*p*-value); * Wilcoxon signed-rank test with Holm adjustment; Δ: Hodges–Lehmann estimator; r: effect size; W: Kendall’s W. NR: Not reported.

**Table 5:** Pelvic floor muscle strength and pelvic organ prolapse staging (POP-Q) over time.

	BE		FU1		FU2				BE vs. FU1		BE vs. FU2		FU1 vs. FU2	
	*n*	%	*n*	%	*n*	%	Global †	W	r	*p * *	r	*p * *	r	*p * *
Oxford Scale	(*n* = 133)		(*n* = 109)		(*n* = 42)		0.135	0.048	0.097	0.472	0.218	0.315	0.218	0.313
None	25	18.80	19	17.43	5	11.90								
Flickers	36	27.07	29	26.61	17	40.48								
Weak	51	38.35	39	35.78	14	33.33								
Moderate	19	14.29	19	17.43	6	14.29								
Good	1	0.75	2	1.83	0	0.00								
Strong	1	0.75	1	0.92	0	0.00								
Cystocele							<0.001	0.164	0.375	<0.001	0.447	0.001	0.323	0.013
None	32	19.63	23	16.91	11	18.64								
Stage I	59	36.20	62	45.59	30	50.85								
Stage II	60	36.81	47	34.56	15	25.42								
Stage III	12	7.36	4	2.94	3	5.08								
Uterine prolapse							0.115	0.037	0.210	0.043	0.213	0.205	0.175	0.205
None	123	75.46	104	76.47	46	77.97								
Stage I	33	20.25	26	19.12	10	16.95								
Stage II	7	4.29	6	4.41	3	5.08								
Rectocele							<0.001	0.166	0.329	<0.001	0.456	0.001	0.247	0.058
None	52	31.90	43	31.62	23	38.98								
Stage I	80	49.08	78	57.35	30	50.85								
Stage II	29	17.79	14	10.29	5	8.47								
Stage III	2	1.23	1	0.74	1	1.69								

Values are presented as *n* (%). BE: baseline evaluation; FU1: first follow-up; FU2: second follow-up. † Friedman test (*p*); * Wilcoxon signed-rank test with Holm adjustment; r: effect size; W: Kendall’s W. Oxford scale categories: None (0), Flickers (1), Weak (2), Moderate (3), Good (4), Strong (5).

**Table 6:** Correlation between pulse energy delivered and clinical outcomes.

	Pulse Energy (J) at First Session				Pulse Energy (J) at Second Session			
	Anterior	Posterior	360°	Vulvar Region	Anterior	Posterior	360°	Vulvar Region
FSFI (Total score)								
r	0.101	0.200	−0.076	−0.036	0.171	-	−0.124	0.228
*p*	0.289	0.800	0.383	0.702	0.240	-	0.354	0.115
Vaginal hiatus (cm)								
r	0.410	0.671	0.092	0.137	0.260	0.527	0.095	0.063
*p*	<0.001	0.215	0.247	0.109	0.005	0.361	0.262	0.490
Vaginal length (cm)								
r	−0.126	0.224	0.205	0.158	−0.114	0.224	0.224	0.264
*p*	0.145	0.718	0.009	0.063	0.223	0.718	0.008	0.003

Values represent Spearman correlation coefficients r and associated *p*-values. FSFI: Female Sexual Function Index.

## Data Availability

Data are available from the corresponding author upon reasonable request because data are not publicly available due to privacy or ethical restrictions.

## Abbreviations

The following abbreviations are used in this manuscript:

BE	Baseline Evaluation
BMI	Body Mass Index
FSFI	Female Sexual Function Index
FU1	First Follow-up
FU2	Second Follow-up
PFD	Pelvic Floor Dysfunction
POP-Q	Pelvic Organ Prolapse Quantification
SD	Standard Deviation
SUI	Stress Urinary Incontinence
